# Enhanced Early Vascularization and Tissue Formation in a Biphasic Collagen Hydrogel Dermal Regeneration Template

**DOI:** 10.1111/wrr.70152

**Published:** 2026-04-19

**Authors:** Derek Luong, Rachael Cohen, Adam Weisel, Ravyn Dorleus, Jonathan Yao, Carol Novotney, Jason A. Spector, Yulia Sapir‐Lekhovitser

**Affiliations:** ^1^ FesariusTherapeutics Inc New York New York USA; ^2^ Veritas Dermatopathology PLLC New York New York USA; ^3^ Center for Animal Resources and Education (C.A.R.E.) Montefiore Medical Center Bronx New York USA; ^4^ Division of Plastic and Reconstructive Surgery, Department of Surgery Weill Cornell Medicine New York New York USA; ^5^ Nancy E. and Peter C. Meinig School of Biomedical Engineering Cornell University Ithaca New York USA

**Keywords:** dermal regeneration, DRT, hydrogel, wound healing

## Abstract

Reconstruction of full‐thickness wounds remains a major clinical challenge. Full‐thickness skin grafts (FTSG) are considered an ideal reconstructive option as they reconstitute both epidermal and dermal layers of skin; however, their use is limited by donor site availability and potential donor site morbidity. Dermal regeneration templates (DRTs) were introduced decades ago to reduce dermal harvesting needs and promote formation of a ‘neodermal’ layer, improving both functional and cosmetic outcomes. However, current DRTs are limited by slow vascularization, typically requiring 2–4 weeks for integration. To address these limitations, we developed DermiSphere *h*DRT, a *hydrogel* dermal regeneration template, a biphasic collagen‐based matrix combining a type I collagen hydrogel with densely packed collagen microspheres. The resulting differential‐density interfaces generate a gradient microstructure that supports efficient cellular infiltration and neovascularization. Using a swine model, we evaluated early host response and vascular invasion into *h*DRT without an overlying graft. By Postoperative Day (POD) 3, *h*DRT was fully integrated with the wound bed and resistant to manual shear, whereas the market‐leading DRT (MLT) showed minimal adherence. Quantitative histology revealed significantly greater cellular and endothelial invasion in *h*DRT, with CD31+ vascular channels penetrating nearly halfway through the construct. By POD 7, *h*DRT generated thicker neodermal tissue with extensive vascular networks extending into new granulation tissue. By POD 10, *h*DRT exhibited organised collagen remodelling and interconnected vertical and horizontal vessel growth, while MLT remained incompletely incorporated. These results demonstrate that *h*DRT's unique microarchitecture accelerates cellular infiltration and neovascularization, enabling faster, more reliable formation of a vascularized neodermal layer for full‐thickness skin repair.

**Abbreviations**
DRT: dermal regeneration template
FTST: full‐thickness skin graft
*h*DRT: *hydrogel* dermal regeneration template
IFU: instruction for use
MLT: market leading DRT
STSG: split‐thickness skin graft

## 1 Introduction

The reconstruction of full‐thickness wounds continues to present a significant challenge for surgeons, as anatomic replacement with full‐thickness skin grafting (FTSG) is constrained by a limited supply and potential morbidity of donor sites. Reconstruction using split‐thickness skin grafts (STSG), which contain an epidermal layer with a varying but often minimal amount of dermis, often leads to secondary wound contraction, suboptimal aesthetics and even an unstable wound where despite initial successful re‐epithelialization, the wound fails to maintain closure and is prone to reopening or breakdown [1, 2, 3].

Dermal regeneration templates (DRTs) were developed several decades ago for the purpose of minimising the amount of dermis taken from donor sites when harvesting STSG [4]. Surgeons quickly recognised that because DRTs regenerate a ‘neodermal’ layer, the resultant reconstructed skin was more robust and resulted in improved functional and aesthetic outcomes [5, 6, 7]. Thus, the combination of DRTs and thin STSGs has become a common approach for the management of full‐ and deep partial‐thickness skin loss, as well as other complex wounds [5, 6].

Despite these advancements, DRTs have notable drawbacks and the most significant among them being the extended time course required for vascularization, which varies between 2 and 4 weeks depending upon the location applied, the condition of the wound bed and patient comorbidities [6, 8, 9]. Even when successful, the lengthy process of vascularization and incorporation requires wound immobilisation and prolonged care. This lengthy timeline places a significant burden on patients and their providers and increases the risk of complications, especially infection [6, 10]. While some efforts to shorten this timeline, such as early or simultaneous grafting, have been occasionally successful, they have rarely shown consistent success or have been largely confined to narrowly defined clinical scenarios [11].

To overcome these limitations, our group developed DermiSphere *h*DRT, a *hydrogel* Dermal Regeneration Template hereafter referred to as *h*DRT, a biphasic biomaterial that features continuous interfaces of differential density created by combining a type I collagen‐based hydrogel with denser type I collagen spheres. The close packing of the microspheres maximises the area of differential density interfaces which drive cell invasion. The presence of the denser spheres within the lower density ‘bulk’ collagen alters the natural collagen fibrillization process of the bulk, and those physical inclusions induce a differentiated fibre bulk matrix with larger fibres and denser fibre bundles around the sphere surfaces than between the spheres. This gradient proved crucial for promoting superior cell invasion and neovascularization [12].

Previous studies have evaluated *h*DRT in wound healing models to determine its regenerative potential. In rodent models, the product significantly accelerated neodermal tissue formation in full‐thickness skin wounds, achieving results within 7 days and significantly faster than the market leading DRT (MLT), hereafter referred to as MLT [13]. Furthermore, in swine models, most homologous to human skin wound healing, this innovative formulation led to successful STSG take even when placed simultaneously [14].

While these earlier studies primarily focused on early or simultaneous skin grafting, the current study explores cellular infiltrate of acute host response and angiogenesis into the *h*DRT without an overlying skin graft. It also focuses on investigation at the earlier stages of healing, seeking to deepen our understanding of the mechanisms that allow subsequent successful performance of the device.

## 2 Materials and Methods

### 2.1 Materials and Reagents

Phosphate‐buffered saline (PBS) (Quality Biological, Gaithersburg, MD), bovine collagen type 1 (Symatese, Chaponost, France), 10% neutral buffered formalin (Azer Scientific, Morgantown, PA), Mepitel (Mölnlycke, Gothenburg, Sweden), Xeroform (Covidien, Dublin, Ireland), Burn cotton (Dermapac, Shelton, CT), Bacitracin (Dynarex, Orangeburg, NY), Tegaderm (3M, Saint Paul, MN). Ioban (3M, Saint Paul, MN), Fluffy undercast padding (Specialist, BSN Medical, Hamburg, Germany), Elastikon (Essity, Stockholm, Sweden) and Custom fitted jacket (Lomir, Montreal, Canada).

### 2.2 Fabrication of *h*DRT

*h*DRT (DermiSphere *h*DRT, FesariusTherapeutics Inc.) were purchased and used according to their instruction for use (IFU). Market‐leading DRT (MLT, Integra Matrix Wound Dressing) was purchased and used according to its IFU [8].

### 2.3 In Vivo Swine Full Thickness Skin Defect Model

#### 2.3.1 Animal Studies

Four 7‐month‐old female Yucatan mini swine (> 20 kg) were purchased from a closed herd at Sinclair Bio Resources, Auxvasse, MO. They were prophylactically treated with Draxxin 2.5 mg/kg SQ or Excede 5 mg/kg upon receipt to prevent respiratory effects of shipping stress. They were acclimated for 1 week prior to procedures. All procedures were conducted in accordance with all Federal and State animal welfare laws, regulations, policies and guidelines, in compliance with the ARRIVE guidelines, and with approval by the Montefiore Medical Center Institutional Animal Care and Use Committee (IACUC) (study protocol #21‐02‐101).

Each animal (*N* = 4) received ten 3 × 3 cm full‐thickness skin wounds on the dorsum. Half of the wounds (*N* = 20) were treated with the *h*DRT (DermiSphere *h*DRT), and the other half were treated with the MLT (Integra Matrix Wound Dressing). Wounds were assessed at 3 different timepoints (3, 7, 10 days; *n* = 5 wounds per group per timepoint). A subset of the wounds unrelated to the work in this study was excluded. Treatments were evenly distributed across wound sites to account for positional effects on healing.

#### 2.3.2 Surgical Procedure

Prior to surgery, swine were pretreated with atropine (0.04 mg/kg) im (15 min) prior to administration of sedatives telazol (4.4 mg/kg im) and ketamine (2.2 mg/kg im). Anaesthesia was induced with propofol (1–3 mg/kg iv) for orotracheal intubation, followed by isoflurane (1%–5%) anaesthesia maintenance with 100% oxygen as carrier gas, flow rate of 2 L/min delivered via ventilator, respiratory rate 5–18/min, tidal volume 10–15 mL/kg, inspiratory time < 1.5 s, ETCO2 35–46 mmHg. A constant intravenous infusion of Lactated Ringers solution or Normosol was given at a rate of 2‐5 mL/kg/h. Vital signs monitoring included SPO2, ETCO2, CRT/MM, jaw tone presence, heart rate and respiratory rate.

Buprenorphine HCL (0.02 mg/kg, im) was used for pre‐emptive analgesia with buprenorphine sustained‐release (0.2 mg sq) used for 72 h postoperative analgesia. Hair was clipped, followed by tattooing the planned wound edges and the skin was prepped with chlorohexidine and ethanol. Aseptic technique was used for all surgical procedures. Full‐thickness skin wounds were made down to the subcutaneous fat using a 10‐blade along the border of the tattoo and haemostasis achieved with gauze and gentle pressure. Following removal of the incised tissue and haemostasis, *h*DRT or MLT products were placed into the wound according to their respective IFUs. Briefly, the *h*DRT sheet was removed from its protective packaging (outer foil pouch and inner blister tray), separated from its carrier mesh and applied directly to the wound bed. MLT was removed from its protective packaging (outer foil pouch and polyethylene backing) and applied directly to the wound bed. Once applied, both DRTs were covered with a Mepitel non‐absorbent dressing (Mölnlycke, Gothenburg, Sweden) and a bulky secondary dressing consisting of Xeroform (Covidien, Dublin, Ireland), bacitracin (Dynarex, Orangeburg, NY) and burn cotton (Dermapac, Shelton, CT) to ensure uniform pressure distribution and optimal contact with the wound bed. All wounds were then wrapped in Ioban (3 M, Saint Paul, MN), followed by cast padding (Specialist, BSN Medical, Hamburg, Germany), Elastikon and a custom fitted jacket (Lomir, Montreal, Canada) to maintain dressing integrity. Upon removal of the bolster (at 7 days POD or sooner if biopsy performed), dressings were changed and wounds were imaged three times per week for the remainder of the experiment.

#### 2.3.3 Explant Procedure and Evaluation of Product Integration Into the Wound Bed

On PODs 3, 7 and 10, strip biopsies measuring 0.5 cm × 4 cm were collected from each experimental group, centred over the wound to include both the entire wound and adjacent normal skin (*N* = 5). Following the removal of excess subcutaneous fat, the excised tissue was examined for evidence of product integration by subjecting it to gentle shear forces to assess the adherence of the product to the underlying tissue. Finally, the excised specimens were fixed in 10% formalin solution for preservation.

#### 2.3.4 Histopathologic Evaluation

All samples were processed by Histowiz Inc. (Brooklyn, NY) using an automated workflow. Tissue samples were processed, embedded in paraffin and sectioned at 5 μm. Samples were stained with either haematoxylin and eosin or picrosirius red (PSR) for evaluation of tissue formation in the DRTs.

#### 2.3.5 Immunohistochemistry

Immunohistochemical staining was performed at Histowiz Inc. (Long Island City, NY), using a Leica Bond RX automated stainer (Leica Microsystems) and a fully automated workflow. Samples were processed, embedded in paraffin and sectioned at 4 μm. The slides were dewaxed using xylene and alcohol‐based dewaxing solutions. Epitope retrieval was performed by heat‐induced epitope retrieval (HIER) of the formalin‐fixed, paraffin‐embedded tissue using citrate‐based pH 6 solution (Leica Microsystems, AR9961) for 20 min at 95°C. The tissues were first incubated with peroxide block buffer (Leica Microsystems), followed by incubation with the rabbit anti‐CD31 antibody (Abcam, ab28364) at a 1:50 dilution for 30 min, followed by DAB rabbit secondary reagents: polymer, DAB refine and haematoxylin (Bond Polymer Refine Detection Kit, Leica Microsystems) according to the manufacturer's protocol. The slides were dried, coverslipped (TissueTek‐Prisma Coverslipper) and visualised using a Leica Aperio AT2 slide scanner (Leica Microsystems) at ×40.

#### 2.3.6 Neodermal Thickness

‘Neodermal’ thickness was defined as the distance from the bottom of the DRT up to the surface of the wound. Ten measurements, uniformly distributed along the length of each wound (*n* = 5 per group) were taken and the average ‘neodermal’ thickness for each specimen was calculated.

#### 2.3.7 Quantification of Cell Invasion (Day 3)

Inflammatory infiltration into the template was assessed according to a previously published methodology [15]. A rectangular region 350 μm in width, extending from the wound bed (deep) to the surface (superficial) encompassing the full thickness of the respective DRT (*h*DRT or MLT), was defined. This rectangle was divided into five equal quintiles. Inflammatory cell infiltrate was quantified for each wound (*n* = 5 per group) by manually counting cells with intact nuclei under ×20 magnification at five randomly picked sections along the wound by a non‐blinded operator within each quintile, with the firsst quintile corresponding to the region closest to the wound bed (deep) and the fifth quintile corresponding to the region nearest to the wound surface (superficial) (Figure S3). The number of cells in each quintile was normalised by the corresponding quintile area, yielding a cell density (cells/mm^2^). An unpaired *t*‐test was used to compare means of each quintile between the groups at each timepoint, with statistical significance set at *p* ≤ 0.05.

#### 2.3.8 Vascularized Tissue Thickness Evaluation

Vascularized tissue was defined as tissue containing clearly identified blood vessels (lumens), as indicated by CD31 expression within the lining of luminal structures. For each wound (*n* = 5 per group), five selected measurements, evenly distributed across the wound, were taken from the wound bed to the farthest detected blood vessel and averaged to determine vascularized tissue thickness of the specimen.

### 2.4 Statistical Analysis

Results are presented as mean ± standard deviations unless otherwise specified. Differences between multiple groups were determined using multiple comparisons testing using the Holm–Šídák test. Significance was set at *p* ≤ 0.05.

## 3 Results

### 3.1 DRT Incorporation

**FIGURE 1:**
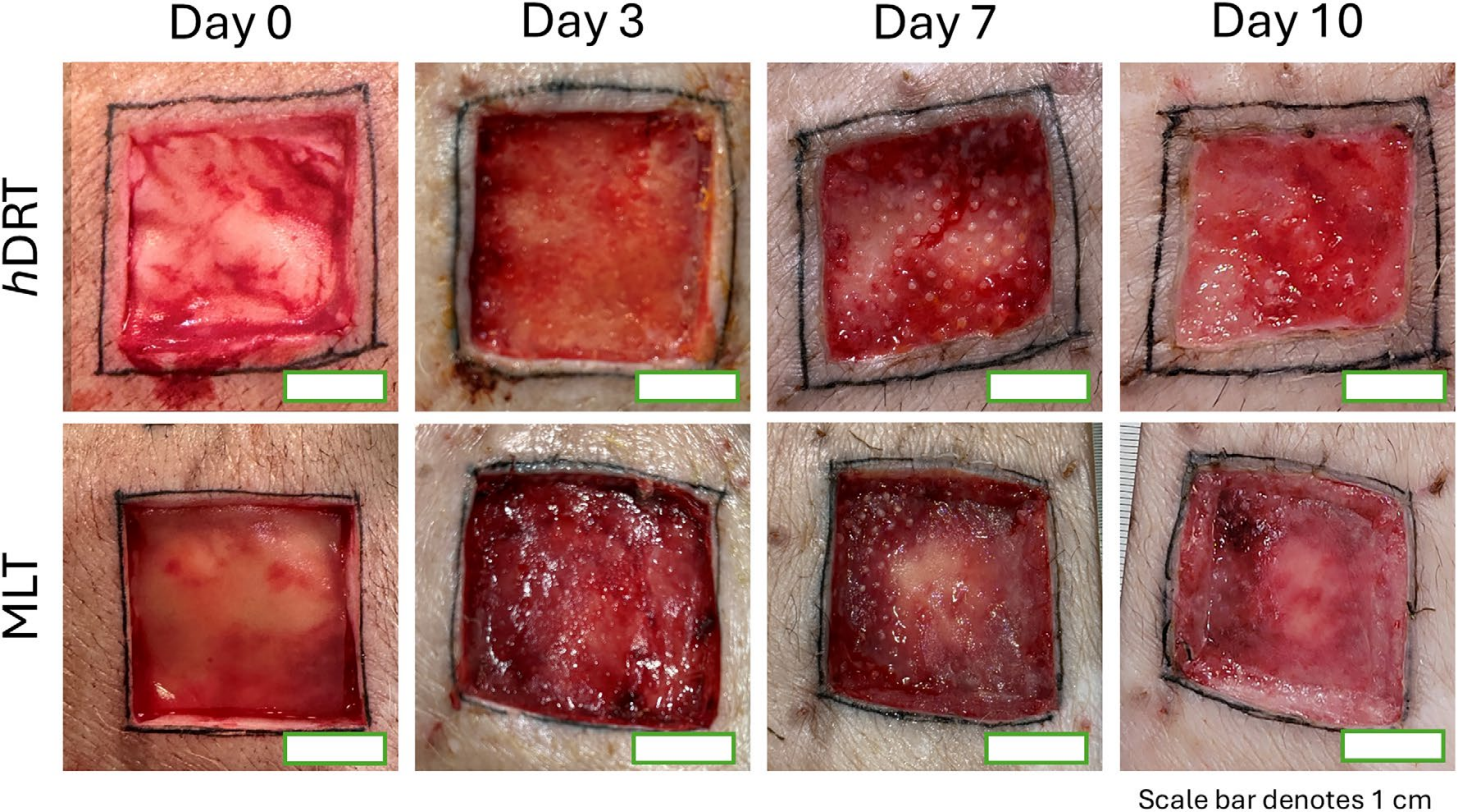
Gross images of wounds at 3, 7 and 10 days post implantation. Scale bar equals 1 cm.

Dermal template incorporation into the wound bed is a critical first step to allow reliable healing and reflects cell invasion into the template and subsequent native tissue deposition. To assess the incorporation of the templates during the study we challenged both experimental groups by subjecting them to gentle manual shear forces (see Section 2.3.3) at harvest. At 3 days (3d) post implantation, the *h*DRT was fully incorporated as demonstrated by its adherence to the wound bed despite gentle shearing forces used while excising a strip biopsy. In contrast, wounds treated with the MLT demonstrated minimal integration into the wound bed, as manifested by poor resistance to minimal shear forces and avulsion of the templates from the wound bed during excisional strip biopsy (Figure 1, Table 1 and Figure S1). By POD 7, 100% of the *h*DRT and 80% of the MLT were adherent and by POD 10 all groups were adherent.

**TABLE 1:** Number of samples that were fully integrated into the wound upon harvesting (as defined by no shearing of the device from the wound upon strip biopsy).

Day	3	7	10
*h*DRT	5/5	5/5	5/5
MLT	0/5	4/5	5/5

### 3.2 Early Stages of Inflammatory Infiltrate and Neovascularization in DRTs (Day 3)

#### 3.2.1 Inflammatory Infiltrate

**FIGURE 2:**
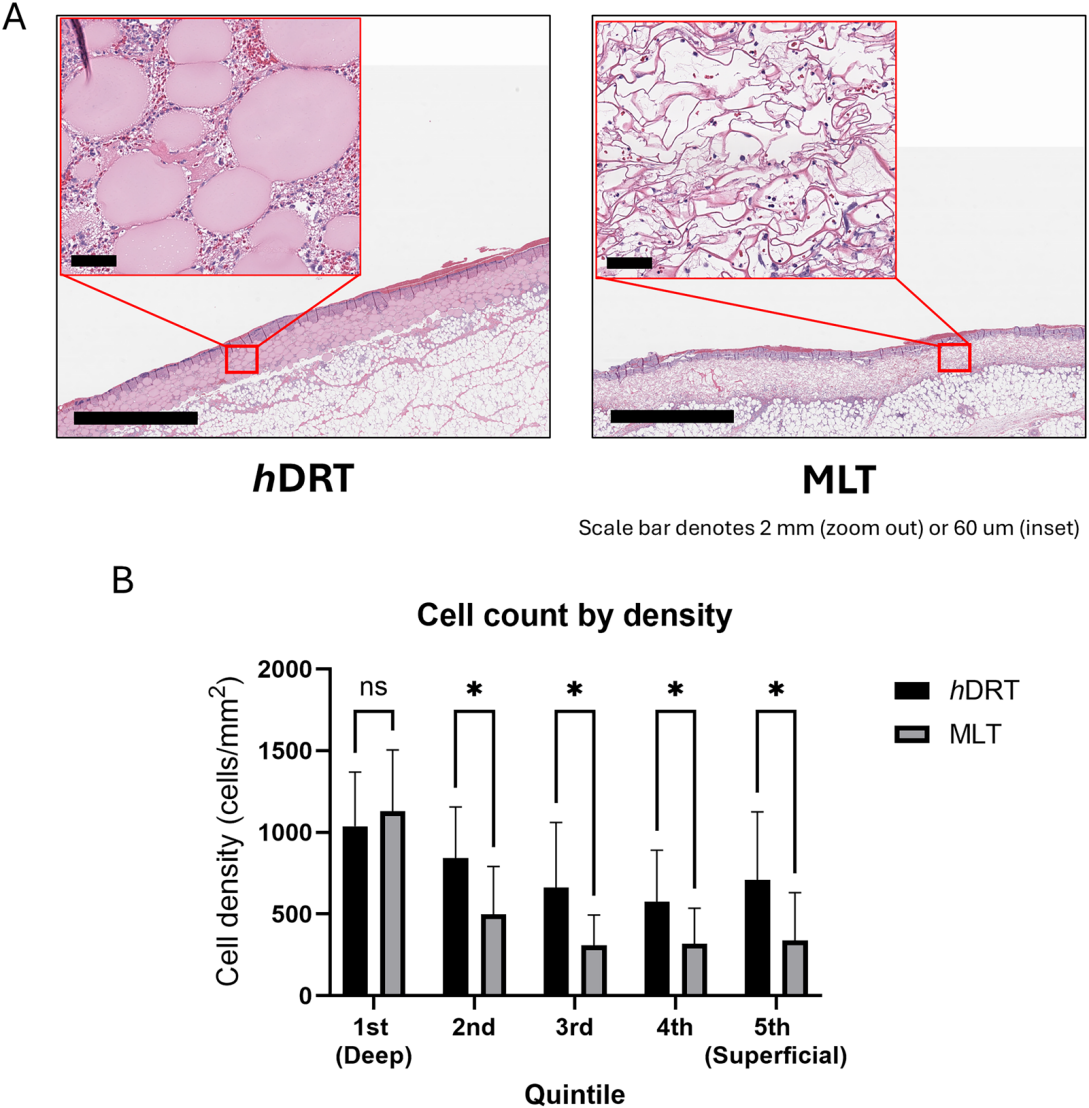
(A) H&E staining of *h*DRT and MLT wounds (*n* = 5 each group) at Postoperative (POD) Day 3 reveals dense cellular infiltration throughout the *h*DRT (insets), whereas there are only sparse cells within MLT. (B) Quantification of cell invasion: at POD 3 cell invasion was significantly higher (* indicates significant difference) into *h*DRT versus MLT within all quintiles beyond the first (deep) quintile (first quintile: *h*DRT 1038 ± 332 cells/mm^2^; MLT 1130 ± 375 cells/mm^2^, *p* = 0.510; second quintile: *h*DRT 840 ± 315 cells/mm^2^; MLT 498 ± 293 cells/mm^2^, *p* < 0.029; third quintile: *h*DRT 663 ± 398 cells/mm^2^; MLT 310 ± 184 cells/mm^2^ *p* = 0.025; fourth quintile: *h*DRT 576 ± 316 cells/mm^2^; MLT 319 ± 217 cells/mm^2^ *p* = 0.038; fifth quintile: *h*DRT 708 ± 417 cells/mm^2^; MLT 338 ± 294 cells/mm^2^ *p* = 0.036).

To better understand the time course of cell invasion into the templates (*n* = 5 per group), we focused our analysis on the earliest time point in the study (Day 3, Figure 2A). The quantity and depth of cell invasion into both templates was quantified using H&E stained cross‐sections (Figure 2B). Cell invasion into *h*DRT was significantly higher when compared to MLT in all quintiles but the first (closest to the wound, ‘deep’) quintile (first quintile: *h*DRT 1038 ± 332 cells/mm^2^; MLT 1130 ± 375 cells/mm^2^, *p* = 0.510; second quintile: *h*DRT 840 ± 315 cells/mm^2^; MLT 498 ± 293 cells/mm^2^, *p* = 0.029; third quintile: *h*DRT 663 ± 398 cells/mm^2^; MLT 310 ± 184 cells/mm^2^ *p* = 0.025; fourth quintile: *h*DRT 576 ± 316 cells/mm^2^; MLT 319 ± 217 cells/mm^2^ *p* = 0.038; fifth (‘superficial’) quintile: *h*DRT 708 ± 417 cells/mm^2^; MLT 338 ± 294 cells/mm^2^ *p* = 0.036).

#### 3.2.2 Neovascularization

**FIGURE 3:**
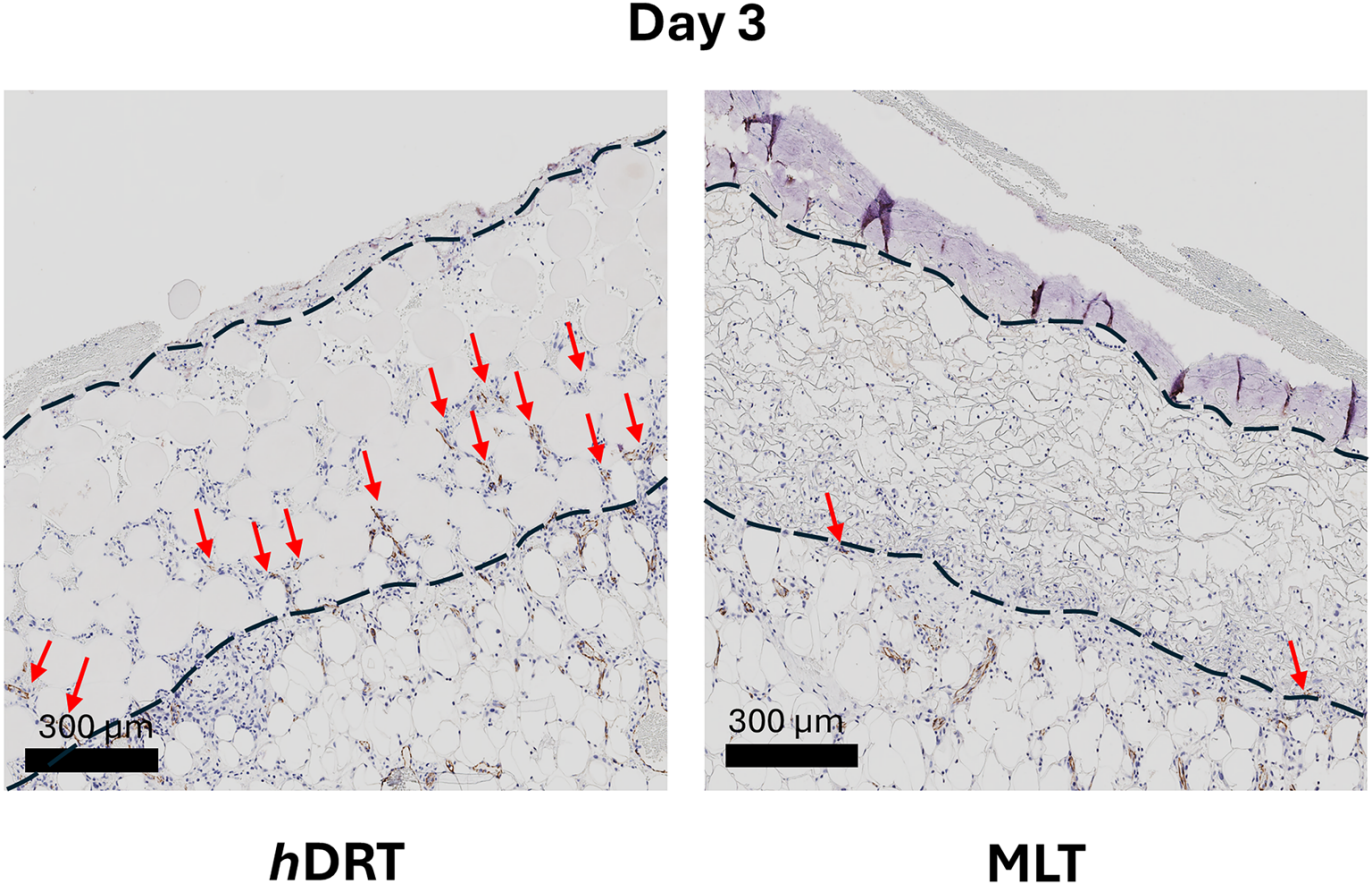
CD31 expression. At POD 3, *h*DRT showed infiltration of CD31+ cells and cell bundles up to halfway through the construct (indicated by red arrows), whereas MLT exhibited sparse invasion limited to the interface with the wound.

By Day 3 post implantation, both individual and bundles of CD31 expressing cells were clearly noted within the *h*DRT group penetrating nearly halfway through thickness of the construct. In contrast, MLT demonstrated sparse CD31+ cells and the few that were seen were present only at the template/wound interface (Figure 3).

### 3.3 Native Tissue Deposition Analysis

#### 3.3.1 Neodermal Thickness (Day 7 and Day 10)

**FIGURE 4:**
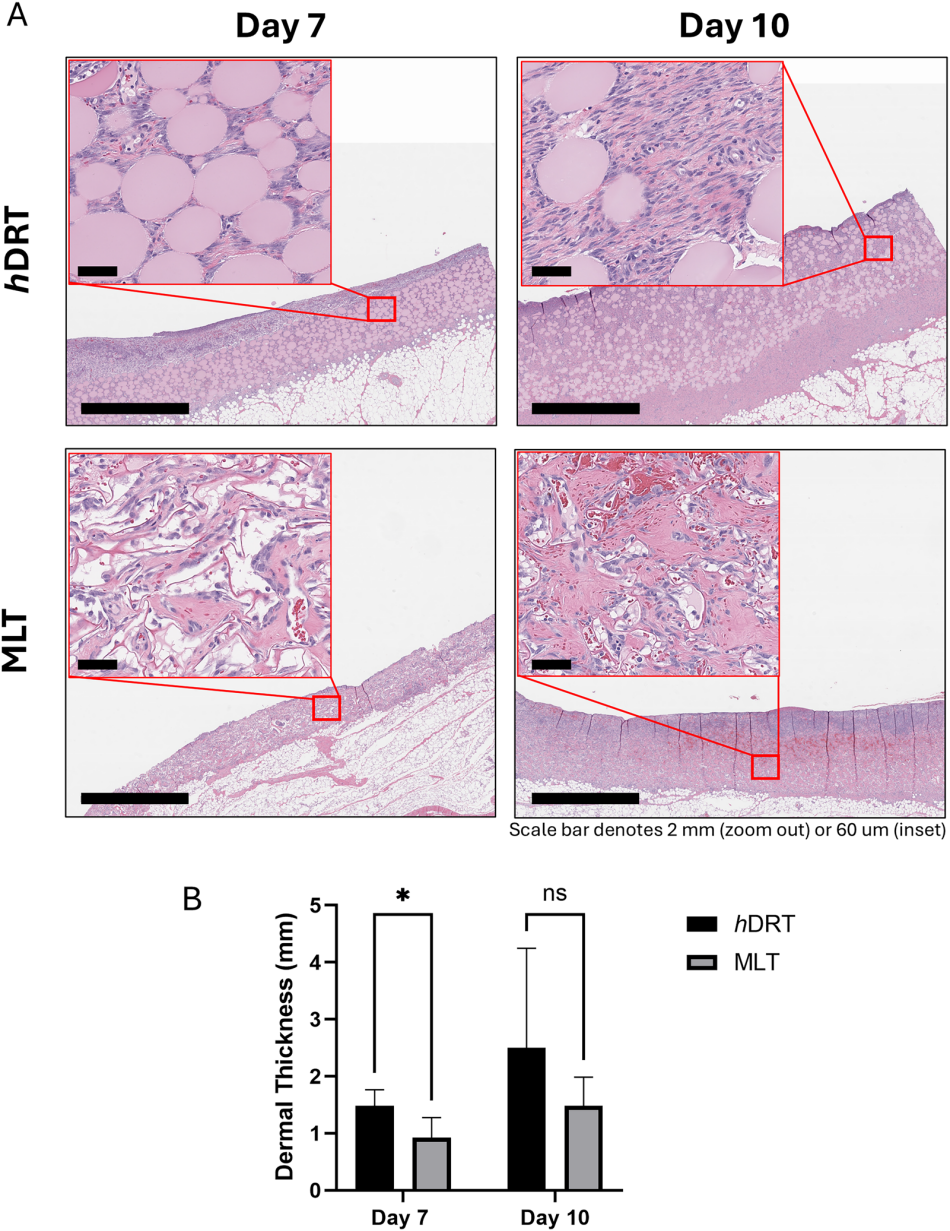
(A) H&E staining of *h*DRT and MLT wounds at POD 7 and 10 demonstrates time course of new tissue deposition for *h*DRT and MLT. (B) Quantification of wound ‘neodermal’ thickness (*n* = 5 each group) for *h*DRT and MLT wounds at POD 7 and 10; *h*DRT generated a thicker (* indicates significant difference) ‘neodermis’ than MLT at Day 7 (1484.8 ± 279.4 vs. 928.4 ± 350.1 mm, *p* = 0.024) and Day 10, with the latter not reaching significance (2501.4 ± 1745 vs. 1485.2 ± 498 mm, *p* = 0.246).

DRTs support deposition of native tissue to replace the lost dermis in full‐thickness wounds. Thus, ‘neodermal’ tissue thickness is often reported as a method to characterise DRT performance. Neither DRT had sufficient neotissue deposition after 3 days for proper measurement (*n* = 5 per group). *h*DRT generated significantly thicker ‘neodermal’ tissue than MLT at Day 7 (1484.8 ± 279.4 vs. 928.4 ± 350.1 mm, *p* = 0.024). On POD 10 a similar but non‐significant difference was observed (2501.4 ± 1745 vs. 1485.2 ± 498 mm, *p* = 0.246) (Figure 4).

#### 3.3.2 Scaffold Vascularization (Day 7 and Day 10)

**FIGURE 5:**
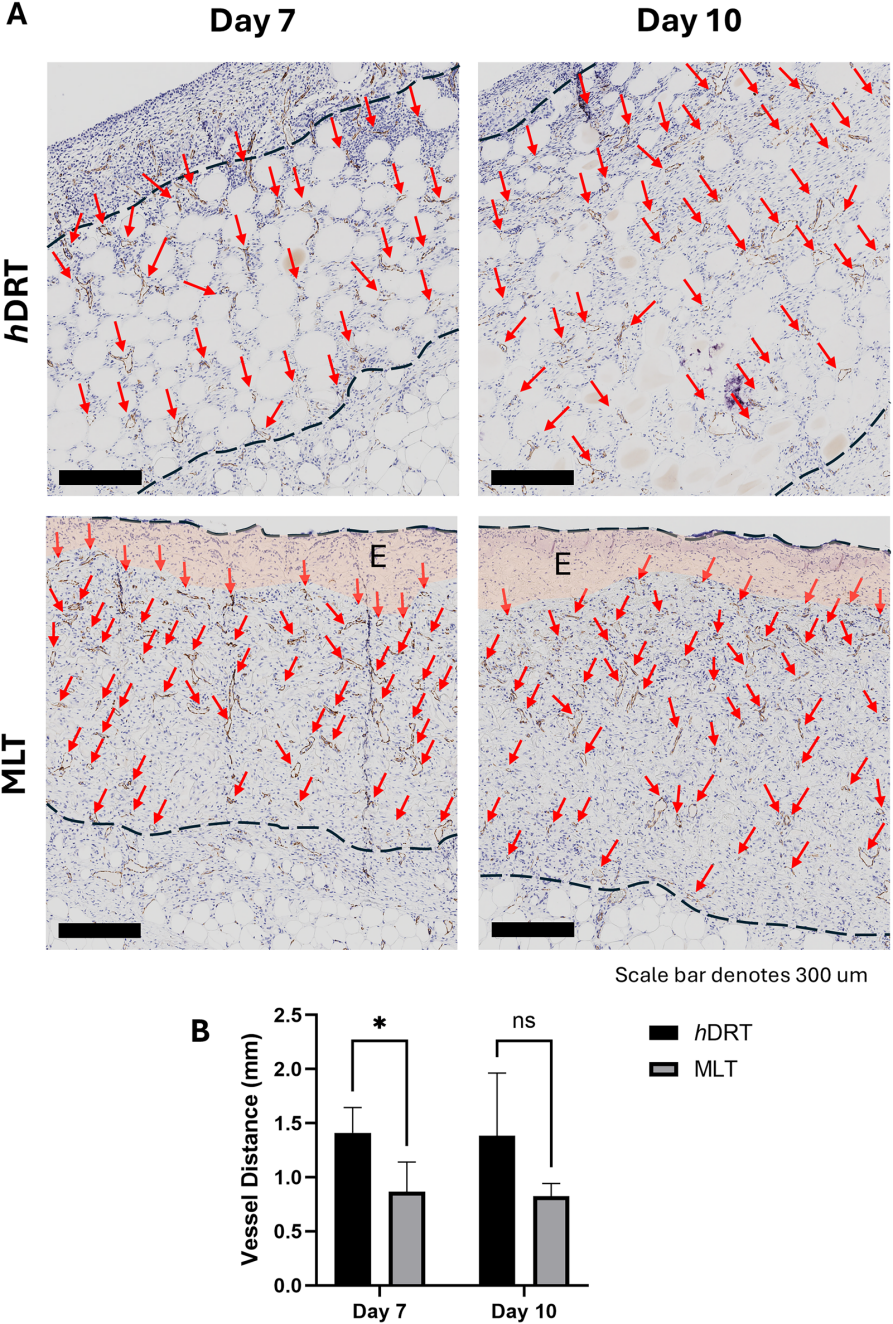
(A) CD31 expression at 7 and 10 days post‐implantation. By Day 7, *h*DRT displayed complete vascularization throughout the construct (new vessels indicated by red arrows), whereas MLT remained devoid of vessels in its uppermost portion (E/empty). (B) Depth of vascular invasion into the DRTs (*n* = 5 each group, * indicates significant difference).

By POD 7 and continuing through POD 10, well‐defined luminal structures expressing CD31 (indicative of endothelial‐lined vasculature) were observed (*n* = 5 per group) throughout *h*DRT. This was accompanied by the development of a vascularized layer of granulation tissue on top of the DRT. In contrast, MLT exhibited delayed and incomplete vascularization at both POD 7 and POD 10 (Figure 5A). Quantification of neovascular invasion into the DRT was performed by measuring the distance of the advancing vascular ‘front’ from the wound interface. At POD 7, *h*DRT demonstrated significantly greater neovascular penetration compared to MLT (*h*DRT 7d: 1.411 ± 0.233 vs. MLT 7d: 0.867 ± 0.274 mm, *p* = 0.046), with vascular structures extending not only within the DRT but also into the overlying granulation tissue.

#### 3.3.3 Collagen Deposition

**FIGURE 6:**
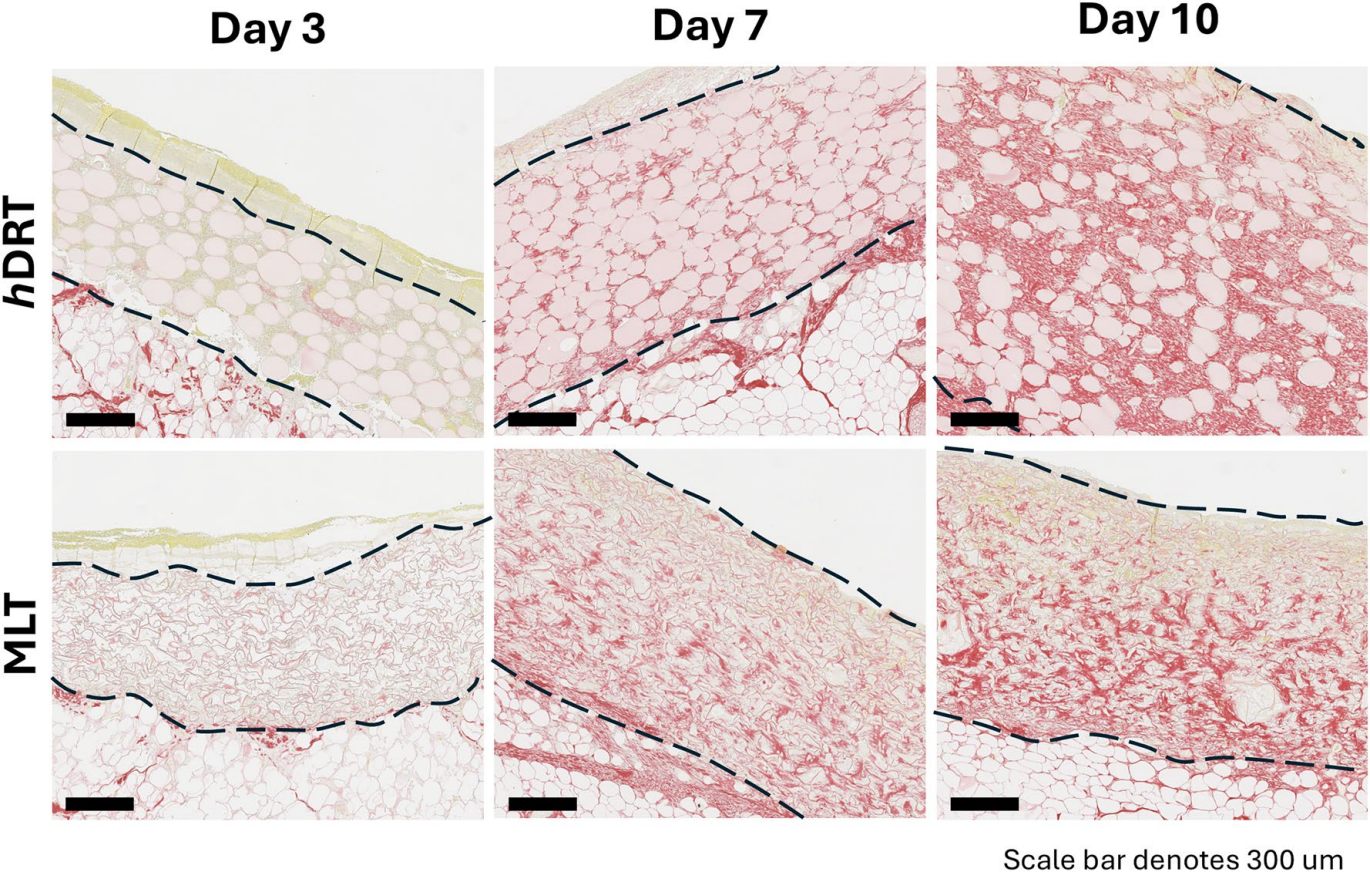
Picrosirius red staining at 3, 7 and 10 days post‐implantation. At Day 3, both *h*DRT and MLT showed minimal evidence of newly deposited collagen, aside from the faint staining of their internal collagen structures (part of template structure). By Day 7, *h*DRT and MLT showed deposition of collagen fibres within their DRT, with *h*DRT showing uniform deposition throughout and MLT lacking deposition in the upper portions of the DRT. At Day 10, collagen had been deposited fairly evenly throughout the *h*DRT. The MLT contained less homogeneous deposition and only sparse new collagen within its uppermost portion. Dashed lines indicate top and bottom of each template.

PSR staining was performed to highlight neocollagen deposition within both templates (Figure 6). Collagen deposition was evident in both templates at Days 7 and 10; however, by POD 10, *h*DRT was almost entirely filled with newly deposited collagen between spheres, while the microspheres remained unchanged. In contrast, MLT exhibited greater void space within its invadable regions at all time points examined.

### 3.4 Qualitative Analysis of Cell Invasion

Histopathological analysis was conducted by an independent board‐certified dermatopathologist (J.Y.). At POD 3, during the early inflammatory phase, distinct differences were observed between the *h*DRT and MLT‐treated wounds. In the *h*DRT group, immune cells, primarily monocytes and neutrophils, migrated into the DRT, concentrating around the microspheres. In contrast, the MLT template had minimal cell infiltration and most of the cells were largely confined to the wound bed/template interface (Figure 2A).

**FIGURE 7:**
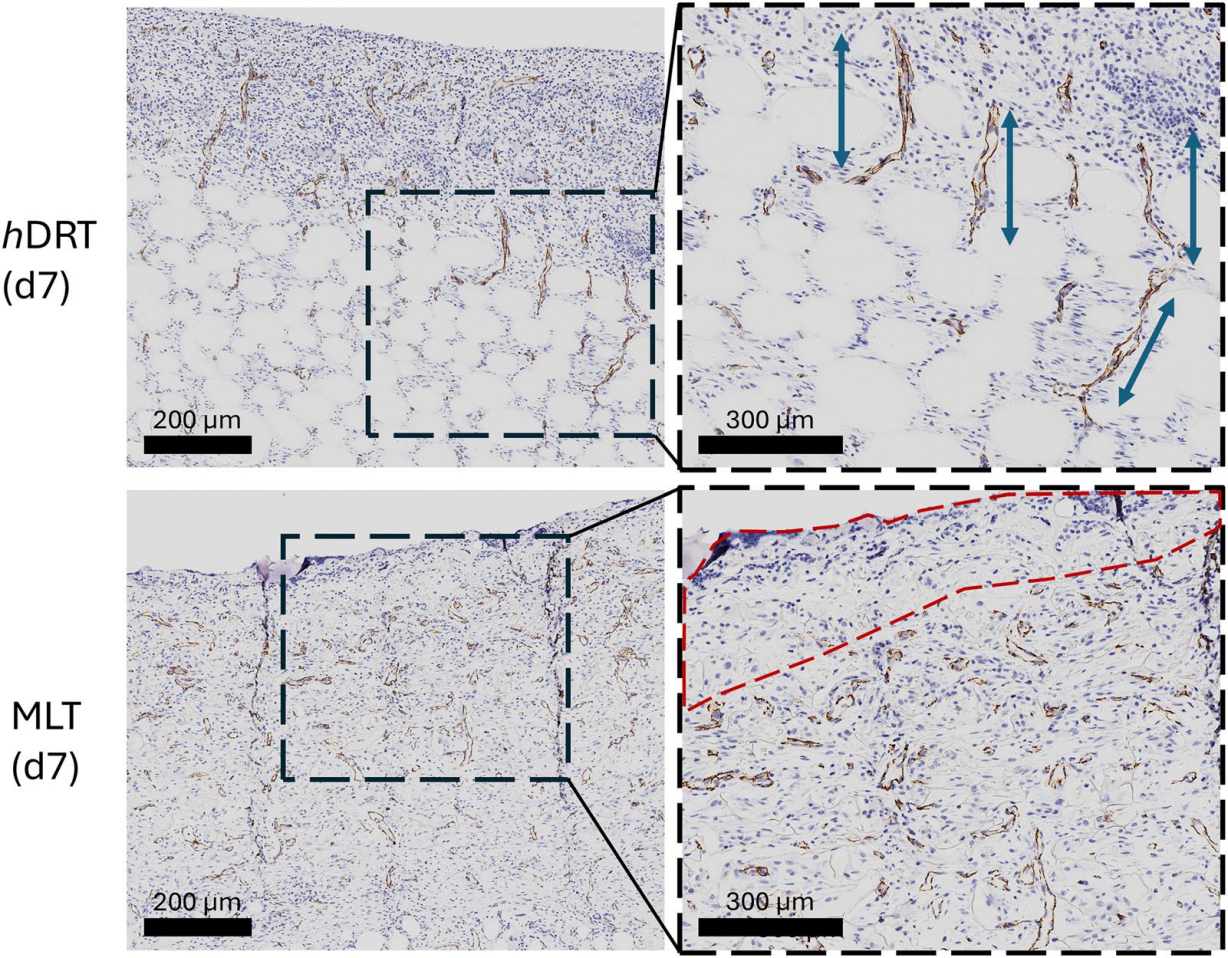
Analysis of CD31 expression revealed distinct differences in vessel orientation and deposition. At Day 7, *h*DRT exhibited vessel formation throughout the construct and into the newly deposited layer of granulation on the surface (above the spheres), with some vessels showing alignment (blue arrows). While MLT demonstrated comparable vascular development, the vessels appeared less organised. Notably, a region lacking vessel staining was observed at the upper portion of the MLT matrix (outlined with red dashes), indicating reduced vascularization further from the wound site.

**FIGURE 8:**
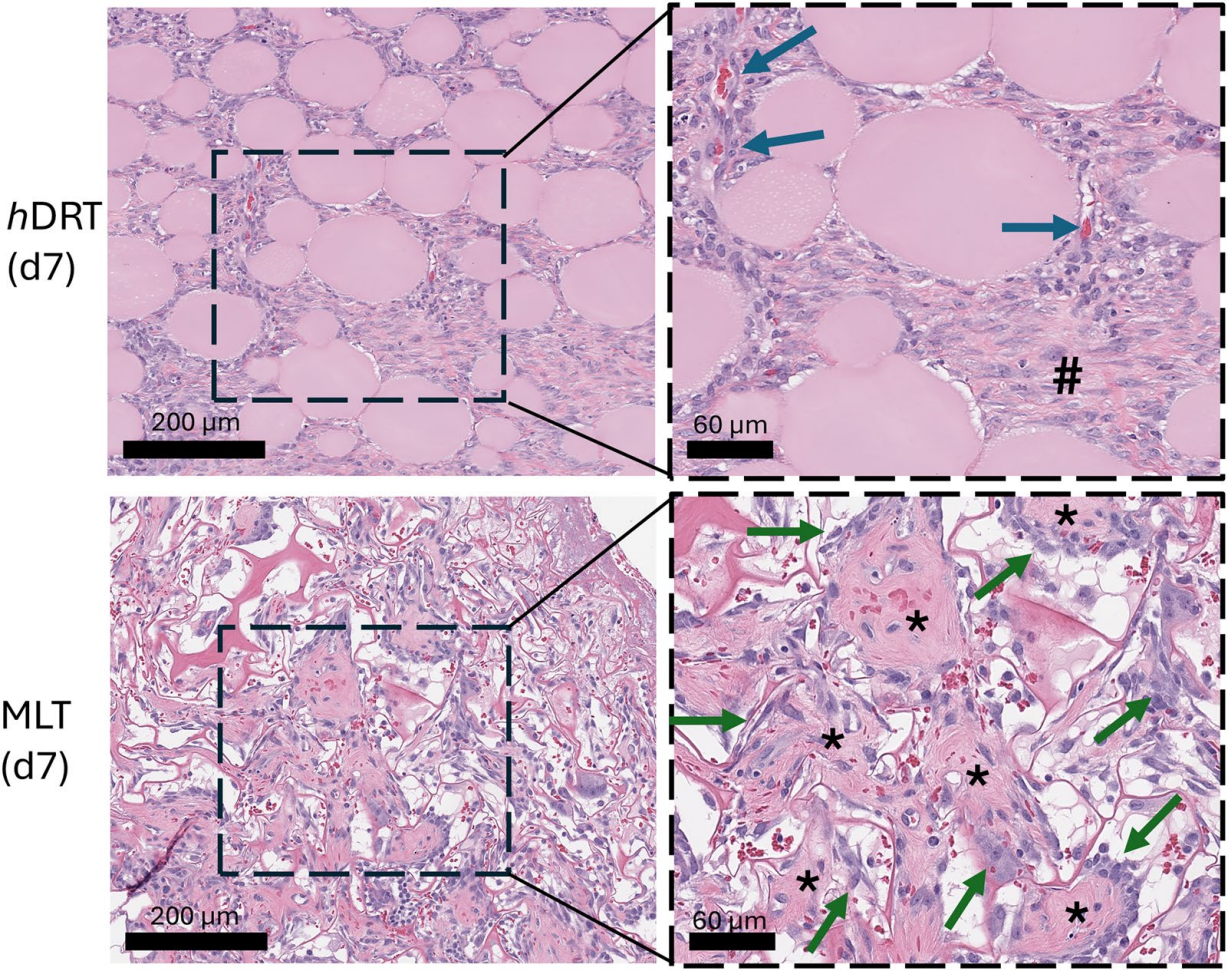
Histologic analysis revealed distinct structural differences in tissue formation between *h*DRT and MLT. By Day 7, *h*DRT exhibited blood vessel formation (blue arrows) along with collagen deposition throughout its structure (#). MLT showed a notable difference in its structural organisation where collagen was deposited within the individual pores of MLT (*) in a ‘basket‐weave’ fashion, which were surrounded by peripheral giant cells (green arrows).

By POD 7, distinct differences in collagen deposition and vascularization were noted between the two DRTs, with the unique microarchitecture of *h*DRT enabling the formation of vertical blood vessels growing upward from the wound bed. The newly deposited granulation tissue on top of *h*DRT appeared thick and well‐vascularized, with vessel networks integrating across vertical and horizontal axes (Figure 7). In contrast, MLT showed a compartmentalised healing pattern. Collagen was deposited inside individual pores in a ‘basket‐weave’ fashion. These isolated collagen units reflected the physical confinement of cells within discrete chambers of the MLT architecture (Figure 7). Blood vessel formation was limited by pore walls, resulting in irregular and non‐vertical vessel growth (Figure 7). Multinuclear giant cells were observed along the inner walls of the pores, further illustrating the restricted immune cell and fibroblast migration within MLT (Figure 8). Because neovascularization had not progressed throughout the thickness of the matrix, no granulation tissue was noted at the top of the matrix.

**FIGURE 9:**
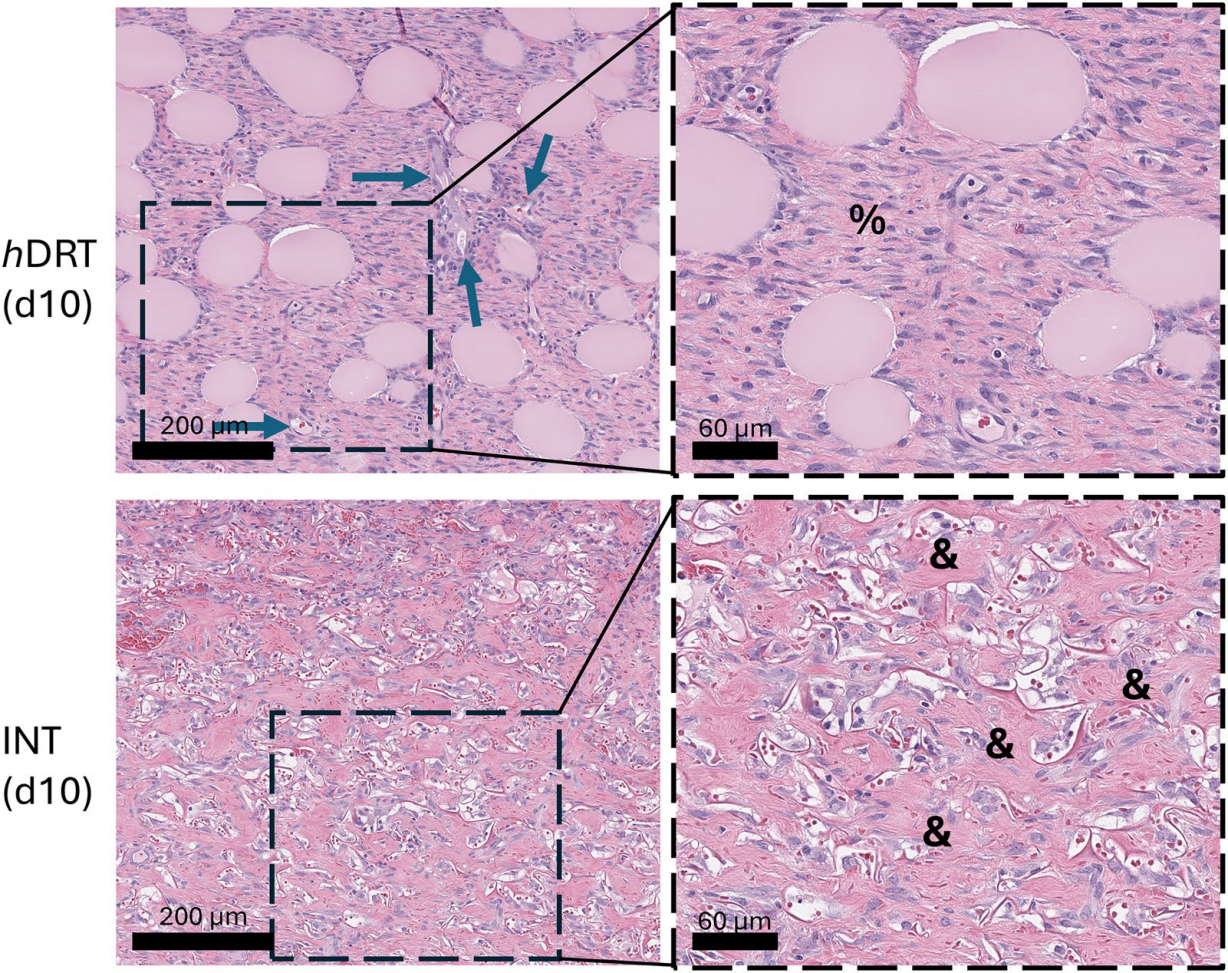
Histologic analysis revealed distinct structural differences in tissue formation between *h*DRT and MLT at later time points. At Day 10, *h*DRT showed continued maturation of collagen (%) and vessel formation (blue arrows). MLT also showed similar development, but this development was limited to individual pores of the MLT, leading to less well organised collagen deposition with more void space within the matrix (&).

By POD 10, collagen maturation is observed. In *h*DRT, collagen was abundant and well‐organised across the DRT, supporting continued vascularization and ‘neodermal’ tissue deposition. The interconnected microarchitecture of *h*DRT facilitated ongoing vertical and lateral vessel formation, maintaining nutrient and oxygen delivery throughout the tissue. Conversely, MLT continued to show poor integration in the upper DRT regions. Collagen deposition was less organised and less extensive, particularly near the DRT surface (Figure 9). Areas of necrosis and cellular debris were apparent, likely due to inadequate perfusion, suggesting issues with vascular access and nutrient supply in those regions.

A summary of these observations is provided in Figure S2.

## 4 Discussion

As the principal interface between the body and external environment, the skin is susceptible to damage from a variety of causes. When damaged, the outermost epidermal layer of skin can regenerate from epithelial stem cells located within the skin appendages. In contrast, the dermis lacks these robust intrinsic regenerative mechanisms and in ‘full‐thickness’ skin wounds, where both the epidermis and dermis are lost, healing eventually occurs through a combination of epithelialization and contraction, with the latter resulting in significant deformity and often functional loss [16, 17]. The gold standard treatment for rapid closure of full‐thickness wounds is skin grafting, where skin, either in its entirety (‐FTSG) or epidermis with a small portion of dermis (STSG), is transplanted to replace lost skin. FTSGs, while preferred for nearly all full‐thickness wounds because they contain a complete dermal layer, are limited in application because of a paucity of sufficient donor sites. STSG, on the other hand, have plentiful donor sites, but their minimal dermal component predictably leads to increased secondary contraction, potential hypertrophy and overall inferior aesthetic and functional results [18].

DRTs were originally developed several decades ago to replace lost dermis by providing a scaffold to support cell infiltration and vascularization to form a neo‐dermis. However, the most widely used DRT in contemporary clinical practice requires 2–4 weeks to achieve sufficient vascularization before it can accept a STSG, thus necessitating two separate surgeries to achieve wound closure [9]. Further, during the 2–4‐week interval before sufficient vascularization is achieved (to allow for coverage with a STSG), there is an increased risk of infection and clinical experience has shown that challenging wound environments (e.g., exposed tendon or hardware) may require even longer intervals to achieve vascularization or they may fail to sufficiently vascularize [9, 19].

To address the limitations of current DRTs, we developed a *h*DRT by embedding type I collagen microspheres in a lower density Type I collagen hydrogel, thus creating interfaces of differential densities that support cellular infiltration within the DRT [12, 13]. In a recent study in a large animal model, we demonstrated that this composite DRT enabled simultaneous implantation with an STSG, effectively eliminating the standard 2–4 week interval required for vascularization [14]. In that study, when compared to a MLT, *h*DRT produced neodermal tissue of comparable thickness and maturity in 2 weeks less time and one fewer surgery than the MLT.

Motivated by these findings, we aimed to explore the underlying mechanisms driving this phenomenon at earlier timepoints and without the possible confounding effect of an overlying STSG (which provides cells and angiogenic factors that may contribute to the wound healing process [20]).

Successful integration of the dermal template into the wound bed is essential for consistent wound healing, as it suggests successful cellular infiltration and the formation of new tissue. As early as POD 3, *h*DRTs were well integrated into the wound bed as evidenced by the fact that when a strip biopsy was harvested from the centre of the wound, the *h*DRT and underlying tissue were easily removed as a composite piece. In contrast, the MLT consistently delaminated upon excision with a scalpel from the underlying tissue, indicating poor integration at this early time point.

These gross observations were supported by histological evidence showing that by Day 3 post‐implantation, *h*DRT had markedly higher levels of cell infiltration than MLT, with greater overall cell density and deeper tissue penetration. Quantitative analysis of cell invasion revealed significantly higher cell counts across all regions of *h*DRT beyond the layer adjacent to the wound bed. These results suggest that the distinctive scaffold architecture of hDRT facilitates enhanced cellular migration into deeper regions of the scaffold compared to MLT. CD31+ expressing cells, often forming clusters or luminal structures, were noted throughout the lower half of the construct only 3 days after implantation, whereas the only CD31+ expressing cells seen within the MLT were limited to the wound‐template interface. Taken together, these data indicate that *h*DRT was more rapidly and effectively incorporating into the wound bed.

To better understand the early differences in cellular and endothelial cell infiltration observed on Day 3 and previously discussed, we examined vascularization of the templates over time. By POD 7, CD31+ expressing luminal structures were apparent not only throughout the entire thickness of *h*DRT but also extended into newly deposited granulation tissue overlying the DRT. In contrast, MLT showed delayed and incomplete neovascularization, with the uppermost portions of the DRT devoid of CD31+ cells/structures. Quantification of vascularized tissue invasion demonstrated that *h*DRT was significantly more vascularized throughout its thickness compared to MLT.

Importantly, qualitative differences in vascular organisation were also observed. Dermatopathologist evaluation determined that vessels within *h*DRT appeared more vertically oriented. In contrast, MLT, with its inherently porous architecture, exhibited a more irregular vascular network formation. These vascularization patterns reflect the influence of DRT microarchitecture on guiding tissue patterning. Although the large pores in the MLT supported cell infiltration and neovascularization, it occurred with less efficiency and consistency in the early stages of healing.

This enhanced vascular integration in *h*DRT was paralleled by more extensive extracellular matrix remodelling. PSR staining revealed progressive collagen deposition in both templates, with *h*DRT being nearly fully filled with newly deposited collagen between the microspheres and throughout its thickness by POD 10 whereas the MLT demonstrated more void space at all points examined. The rapid cellular and vascular invasion observed in *h*DRT discussed above contributed to the increase in neodermal tissue formation seen; by Day 7, *h*DRT showed significantly greater deposition of neodermal tissue (thickness) when compared to MLT.

Collectively, these findings provide insight into how *h*DRT consistently supported ‘take’ of STSG placed simultaneously with *h*DRT into a full‐thickness wound, as was observed in earlier studies [14]. Although much more rapidly invaded with endothelial precursors than MLT, the *h*DRT does not contain vessels throughout its thickness by POD 3. It has been shown that cells within a STSG must reestablish a connection with the recipient's blood supply (inosculation) within 48–72 h to survive [21, 22]. However, the consistent survival of STSG over *h*DRT suggests that the demonstrated degree of vascular invasion is sufficient. Taken together, these findings provide some mechanistic insight into previous in vivo studies supporting early or even simultaneous use of *h*DRT with STSG.

## 5 Conclusions

These results provide insight into how *h*DRT overcomes the limitations of conventional DRTs by supporting simultaneous STSG implantation as seen in our previous work, thus eliminating the need for a delayed second procedure. This advancement holds significant potential for improving wound healing outcomes, particularly in challenging clinical scenarios where rapid and stable engraftment is critical.

## 6 Study Limitations

While this study yields important insights, several limitations should be considered in the interpretation of its findings. In particular, the intrinsic macroscopic and microscopic structural differences between *h*DRT and MLS limited the feasibility of blinding observers taking measurements/performing comparative analyses. During post‐excision shear testing of the DRTs, the evident visual and mechanical disparities between the two materials precluded effective blinding. Likewise, in the histopathological analysis, although the evaluating pathologist was not provided with sample identifiers, the distinct architectural characteristics of *h*DRT and MLS made full blinding challenging. To mitigate potential bias, histologic evaluation was performed by a trained dermatopathologist using standardised scoring criteria. This limitation similarly extended to the quantification of cellular invasion. Finally, future studies incorporating direct comparisons with a broader range of commercially available devices intended for similar use would further contextualise these findings and strengthen conclusions regarding the relative performance of *h*DRT.

## Funding

This work was supported by the National Institutes of Health, 1R44AR082787‐01A1.

## Conflicts of Interest

J.A.S. has a patent on ‘Tissue Scaffold Materials for Tissue Regeneration and Methods of Making’ (US20160287755A1) upon which this technology discussed is based. J.A.S. is a founding shareholder/Senior Medical Officer of FesariusTherapeutics Inc., a start‐up company that has licenced the technology described herein. D.L., A.W., R.C. and Y.S.‐L. are employees of and equity holders in FesariusTherapeutics Inc. The other authors declare no conflicts of interest.

## Supporting information


**Figure S1:** Video (A) and picture (B) of MLT group at POD 3 showing delamination of the template upon harvest of a strip biopsy of the MLT.

**Figure S2:** Summary of dermatopathological analysis.

**Figure S3:** Illustration of cell counting method.

## Data Availability

The data that support the findings of this study are available from Fesarius Therapeutics. Restrictions apply to the availability of these data, which were used under license for this study. Data are available from the author(s) with the permission of Fesarius Therapeutics.
