# Supplementary figures and images for: Enhanced Early Vascularization and Tissue Formation in a Biphasic Collagen Hydrogel Dermal Regeneration Template
Source: Wound Repair Regen. 2026 Apr 19;34(2):e70152. doi: 10.1111/wrr.70152 (PMC13092782; doi:10.1111/wrr.70152)

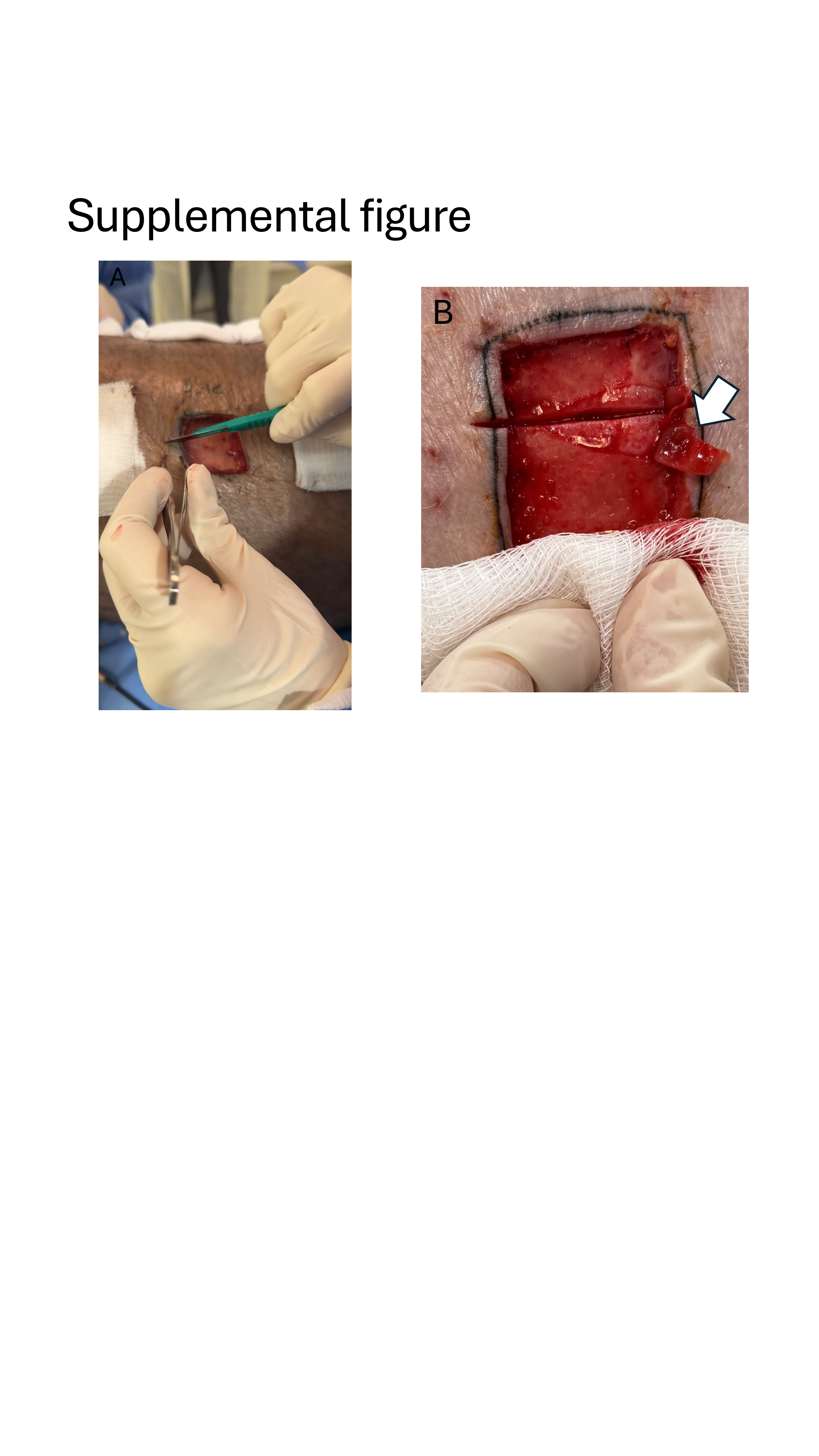

Supplement: Supplementary file 1 — Figure S1: Video (A) and picture (B) of MLT group at POD 3 showing delamination of the template upon harvest of a strip biopsy of the MLT. [file WRR-34-0-s001.tif]

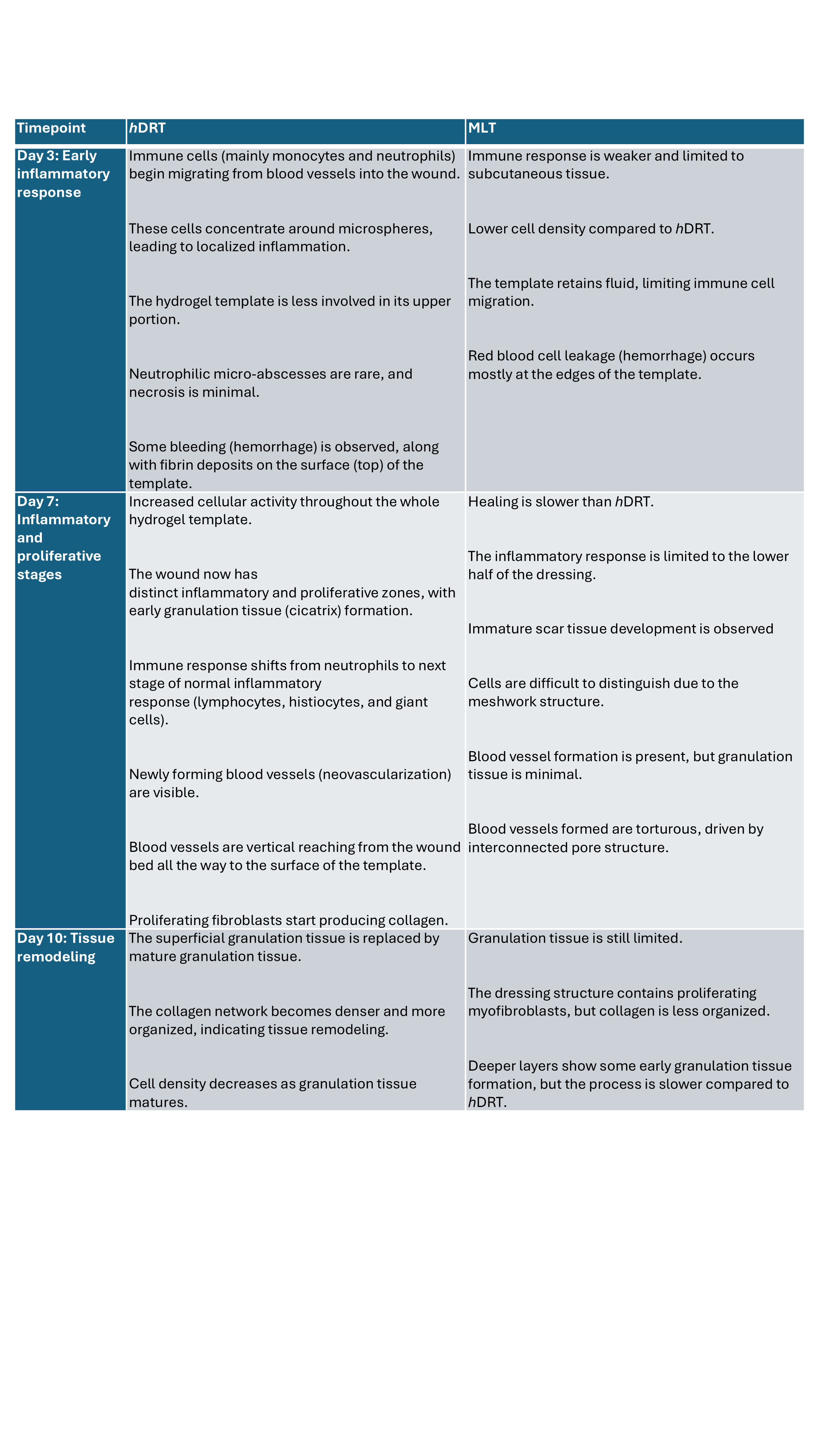

Supplement: Supplementary file 2 — Figure S2: Summary of dermatopathological analysis. [file WRR-34-0-s003.tif]

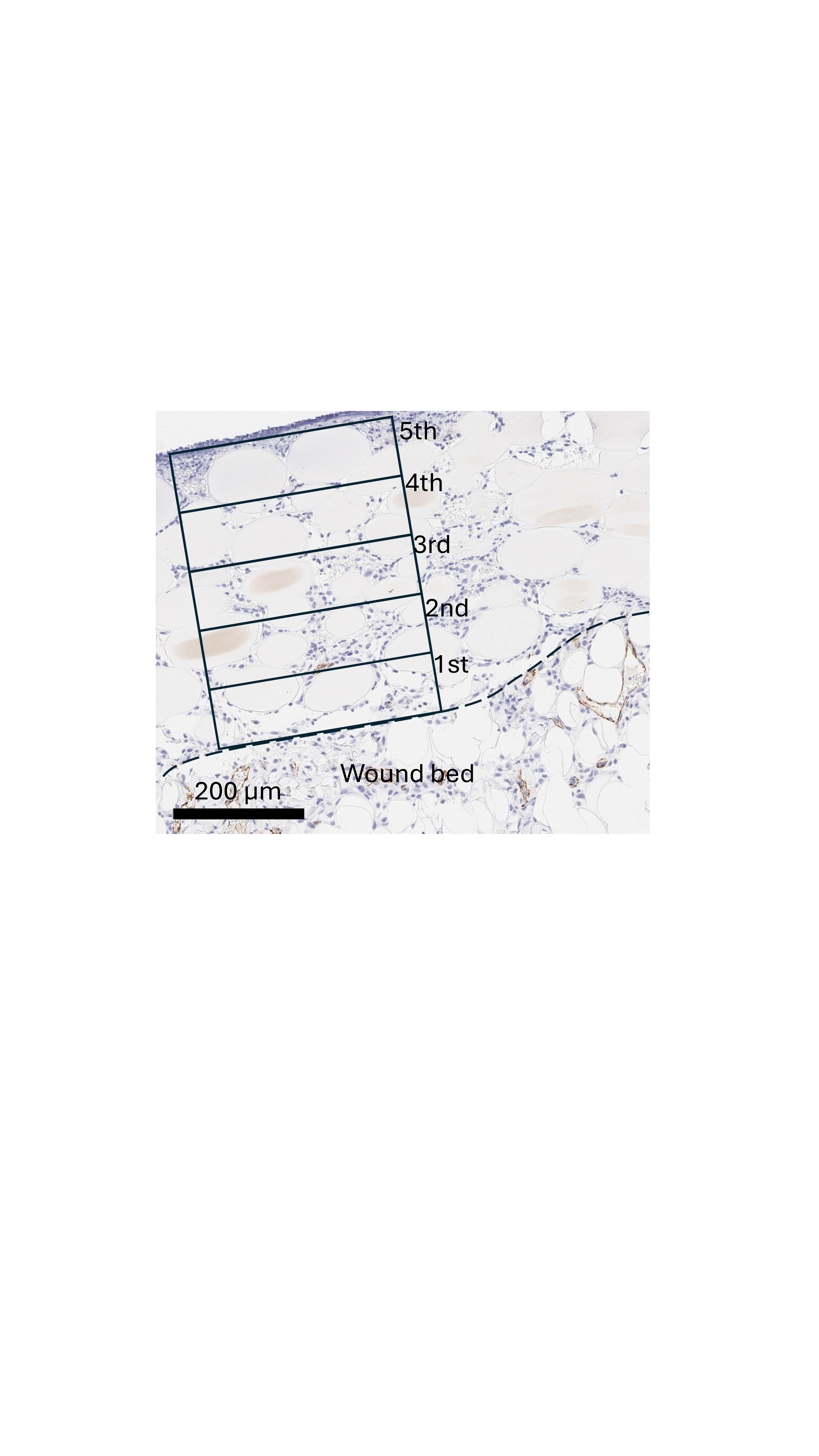

Supplement: Supplementary file 3 — Figure S3: Illustration of cell counting method. [file WRR-34-0-s002.tif]
